# Supplementary material for: ESBL-Type and AmpC-Type Beta-Lactamases in Third Generation Cephalosporin-Resistant Enterobacterales Isolated from Animal Feces in Madagascar
Source: Animals (Basel). 2024 Feb 27;14(5):741. doi: 10.3390/ani14050741 (PMC10931431; doi:10.3390/ani14050741)
Supplement: Supplementary file 1 [file animals-14-00741-s001.zip › Table S1_Sampling dates and locations Enterobacterales Animals Madagascar.pdf]

Supplementary Table S1: Details on the sampling dates and exact locations of the swabbing of the stool droppings.

Sample I.D.	Animal species	Sampling date (year-month-day)	latitude	longitude
ET01	*Echinops telfairi*	2017-03-10	-24.032913	43.721908
HC35	*Macronycteris commersoni*	2017-03-04	-24.045841	43.753921
HC36	*Macronycteris commersoni*	2017-03-04	-24.045841	43.753921
HC37	*Macronycteris commersoni*	2017-03-06	-24.045841	43.753921
HC38	*Macronycteris commersoni*	2017-03-06	-24.045841	43.753921
HC39	*Macronycteris commersoni*	2017-03-08	-24.045841	43.753921
HC40	*Macronycteris commersoni*	2017-03-08	-24.045841	43.753921
MG11r	*Microcebus griseorufus*	2017-02-23	-24.024776	43.736216
MG16r	*Microcebus griseorufus*	2017-04-06	-24.028973	43.739648
MG23r	*Microcebus griseorufus*	2017-03-08	-24.033346	43.721355
MG59	*Microcebus griseorufus*	2017-02-10	-24.022742	43.728882
MG60	*Microcebus griseorufus*	2017-02-10	-24.017216	43.728793
MG61	*Microcebus griseorufus*	2017-02-10	-24.016828	43.728565
MG62	*Microcebus griseorufus*	2017-02-10	-24.016622	43.728481
MG63	*Microcebus griseorufus*	2017-02-10	-24.016752	43.728757
MG64	*Microcebus griseorufus*	2017-02-10	-24.016844	43.728701
MG65	*Microcebus griseorufus*	2017-02-11	-24.02291	43.729003
MG66	*Microcebus griseorufus*	2017-02-11	-24.022742	43.728882
MG67	*Microcebus griseorufus*	2017-02-11	-24.022189	43.72875
MG68	*Microcebus griseorufus*	2017-02-11	-24.021337	43.728384
MG70	*Microcebus griseorufus*	2017-02-11	-24.016715	43.728496
MG71	*Microcebus griseorufus*	2017-02-12	-24.022189	43.72875
MG74	*Microcebus griseorufus*	2017-03-08	-24.030371	43.724834
MG75	*Microcebus griseorufus*	2017-03-08	-24.030181	43.72336
MG76	*Microcebus griseorufus*	2017-03-08	-24.030392	43.721891
MG77	*Microcebus griseorufus*	2017-03-09	-24.033346	43.721355
MG79	*Microcebus griseorufus*	2017-03-09	-24.030099	43.723119
MG84	*Microcebus griseorufus*	2017-03-22	-24.026611	43.737068
MG86	*Microcebus griseorufus*	2017-03-27	-24.026611	43.737068
MG87	*Microcebus griseorufus*	2017-03-27	-24.037652	43.742995
MG88	*Microcebus griseorufus*	2017-03-27	-24.046558	43.699368
MG89	*Microcebus griseorufus*	2017-03-28	-24.034932	43.742667
MG90	*Microcebus griseorufus*	2017-04-04	-24.027832	43.738939
MG91	*Microcebus griseorufus*	2017-04-04	-24.028227	43.739164
MG92	*Microcebus griseorufus*	2017-04-04	-24.032257	43.741946
MG93	*Microcebus griseorufus*	2017-04-05	-24.028973	43.739648
MG94	*Microcebus griseorufus*	2017-04-05	-24.030024	43.740874
MG95	*Microcebus griseorufus*	2017-04-07	-24.025434	43.738611
MG96	*Microcebus griseorufus*	2017-04-07	-24.026568	43.738496
PA64	*Pyxis arachnoides*	2017-03-23	-24.027612	43.706747
RR25	*Rattus rattus*	2017-02-28	-24.078624	43.6997831
RR26	*Rattus rattus*	2017-03-11	-24.026611	43.737068
RR27	*Rattus rattus*	2017-03-12	-24.0427134	43.6947647
RR28	*Rattus rattus*	2017-03-15	-24.079488	43.674248
RR29	*Rattus rattus*	2017-03-23	-24.024521	43.710638
RR30	*Rattus rattus*	2017-04-05	-24.033092	43.739572
SS01	*Setifer setosus*	2017-03-08	-24.032369	43.723176
SS02	*Setifer setosus*	2017-03-14	-24.080277	43.674158
TM110	*Triaenops menamena*	2017-03-03	-24.045841	43.753921

I.D. = identity.
